# Iron from a submarine source impacts the productive layer of the Western Tropical South Pacific (WTSP)

**DOI:** 10.1038/s41598-018-27407-z

**Published:** 2018-06-13

**Authors:** Cécile Guieu, Sophie Bonnet, Anne Petrenko, Christophe Menkes, Valérie Chavagnac, Karine Desboeufs, Christophe Maes, Thierry Moutin

**Affiliations:** 1 Sorbonne Université, CNRS, Laboratoire d’Océanographie de Villefranche, LOV, F-06230 Villefranche-sur-mer, France; 2 The Center for Prototype Climate Modeling, New York University in Abu Dhabi, Abu Dhabi, UAE; 3 Aix Marseille Université, CNRS/Institut National des Sciences de l’Univers, Université de Toulon, Institut de Recherche pour le Développement, Observatoire des Sciences de l’Univers Pythéas, Mediterranean Institute of Oceanography (MIO), F-13288 Marseille, France; 4 LOCEAN, Sorbonne Université, IRD/CNRS/MNHN, IRD, BP A5, 98848 Nouméa Cedex, New Caledonia; 5 Géosciences Environnement Toulouse, CNRS/UPS/IRD/CNES, Université de Toulouse, F-31400 Toulouse, France; 6 Laboratoire Interuniversitaire des Systèmes Atmosphériques (LISA), IPSL, Université Paris Est Créteil (UPEC), Université Paris Diderot (UPD), Créteil, France; 7 IRD/LOPS, IFREMER, CNRS, IUEM, University of Brest, Brest, France

## Abstract

In the Western Tropical South Pacific, patches of high chlorophyll concentrations linked to the occurrence of N_2_-fixing organisms are found in the vicinity of volcanic islands. The survival of these organisms relies on a high bioavailable iron supply whose origin and fluxes remain unknown. Here, we measured high dissolved iron (DFe) concentrations (up to 66 nM) in the euphotic layer, extending zonally over 10 degrees longitude (174 E−175 W) at ∼20°S latitude. DFe atmospheric fluxes were at the lower end of reported values of the remote ocean and could not explain the high DFe concentrations measured in the water column in the vicinity of Tonga. We argue that the high DFe concentrations may be sustained by a submarine source, also characterized by freshwater input and recorded as salinity anomalies by Argo float *in situ* measurements and atlas data. The observed negative salinity anomalies are reproduced by simulations from a general ocean circulation model. Submarine iron sources reaching the euphotic layer may impact nitrogen fixation across the whole region.

## Introduction

The Western Tropical South Pacific (WTSP) Ocean has recently been identified as a hotspot of N_2_ fixation^1^, the main external source of new fixed nitrogen (N) to the surface ocean, which controls primary productivity and carbon export^2,3^. N_2_-fixing organisms (or diazotrophs) have high iron (Fe) quotas relative to non-diazotrophic phytoplankton^4,5^ and the success of diazotrophs in this region has been assumed to be due to the alleviation of Fe limitation^1^. As a result, there is considerable interest in studying and characterizing Fe sources in the WTSP in order to confirm or rule out this hypothesis. In the WTSP, Fe could be supplied by runoff from islands in the vicinity of Melanesian archipelagos^6^ or by atmospheric volcanic inputs as the WTSP includes the Tonga and Vanuatu volcanic arcs. However, these potential Fe sources have not been quantified to date, even though it has recently been shown that volcanic aerosols facilitate natural Fe ocean fertilization around Iceland and the Mariana back-arc^7,8^. Recent numerical computer modeling has shed light on the crucial role played by hydrothermal Fe sources in global biogeochemical budgets, as these submarine sources are a major controlling factor in the column inventory of Fe in ~25% of the ocean^9^. Despite the facts that the WTSP comprises the most active zone for submarine volcanic activity in the world ocean^10^ and that it has been identified as a priority zone to distinguish the trace metal sources of submarine origin from other sources^11,12^, our knowledge on Fe sources in the WTSP remains fragmentary as only a very few dissolved Fe (DFe) data are available within the Melanesian region^13^.

**Figure 1:**
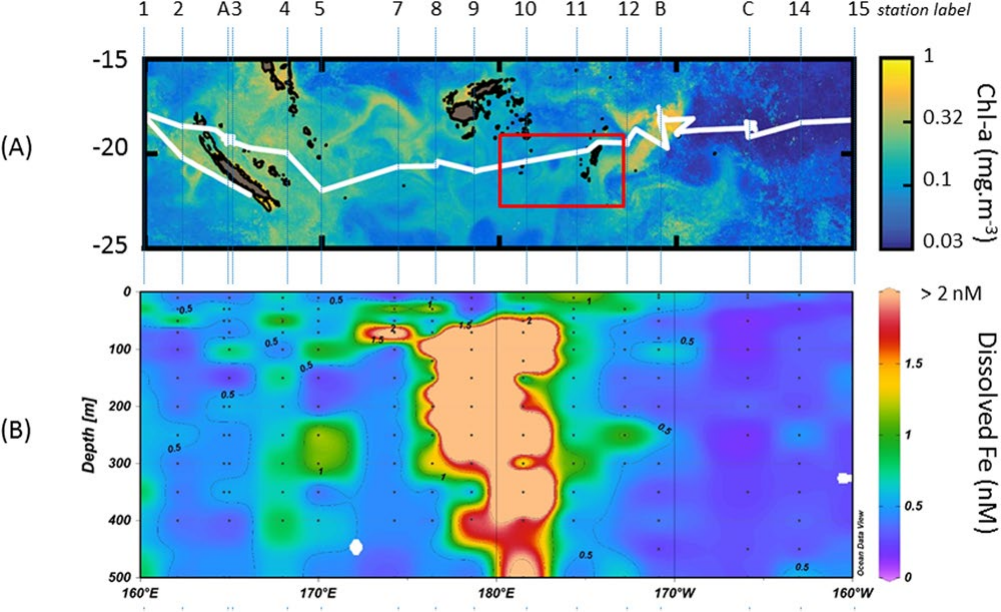
Surface Chlorophyll-a concentration (mg m^−3^) during the 45-day transect of the OUTPACE cruise (**A**) (The ocean color satellite products are produced by CLS. Figure courtesy of A. De Verneil). (**B**) Cross-section of dissolved Fe nM (0–500 m) (full data set in Supplementary Table 2). The box in 1 A shows the area of the ARGO float journey mapped in Fig. 2A.

Here, we quantify the different Fe external sources (atmospheric and submarine) across a 4000-km zonal transect at ~19°S latitude in the WTSP (OUTPACE cruise, http://dx.doi.org/10.17600/15000900, Fig. 1A). We show that a shallow submarine source impacts DFe concentrations up to the productive layer (~0–140 m), where average DFe was as high as 3.8 nM, contrasting radically with the <0.3 nM DFe in the adjacent ultra-oligotrophic South Pacific Gyre. Our results reveal that hydrothermal submarine inputs would explain these high DFe concentrations, rather than atmospheric deposition.

## Results

### Atmospheric source of iron

Atmospheric sources of DFe were suspected to be located west of 170°E longitude, associated with the permanently active Vanuatu volcanoes, and near the Tonga archipelago (175°W longitude). In particular, the Hunga Tonga-Hunga Ha’apai volcano within the Tonga-Kermadec arc erupted between December 19, 2014 and January 24, 2015, before our cruise (February-March 2015). This intense eruption led to the formation of an island whereby volcanic material was emitted into the atmosphere^14^. Total Fe concentrations in our aerosols (Methods and Supplementary Table 1) ranged between 0.013 and 1.96 nmol Fe m^−3^. Atmospheric Fe being associated with ash or dust, Fe fluxes were estimated using a deposition velocity of 1.4 cm. s^−1^ that is typical of coarse particles^15^. These total Fe fluxes were low (15 to 2361 nmol Fe m^−2^ d^−1^) and of the same order of magnitude as the mean Fe fluxes in the non-volcanic remote areas of the South Indian Ocean^16^. Indeed, aerosols collected few weeks after the end of the eruption were likely not impacted by emitted particles that certainly moved quickly out of the region, as previously observed for an eruption of the same volcano in 2009^17^. Derived DFe atmospheric inputs to the water column (Supplementary Table 1) ranged from 0.0007 to 0.04 nmol DFe m^−3^, leading to DFe atmospheric fluxes ranging from 0.85 to 48.7 nmol DFe m^−2^ d^−1^ (0.017 to 0.99 mg DFe m^−2^ yr^−1^). These DFe fluxes, documented in the WTSP for the first time, are at the lower end of values reported for the remote ocean^18^ and support model estimates for dissolved Fe deposition to the WTSP (0.16 to 0.315 mg DFe m^−2^ yr^−1^^18^). Considering a maximum DFe atmospheric flux of 50 nmol m^−2^ d^−1^ depositing for 40 days (duration of the eruption) and impacting a 20-meter-thick surface mixed layer, the DFe concentration in seawater would have been increased by 0.1 nM. As a result, atmospheric inputs could not explain the DFe concentrations (average up to 3.8 nM) measured in the water column west of Tonga in March 2015.

During the Dec. 2014–Jan. 2015 eruption, satellite images have shown release of volcanic particles directly in the ocean by the newly formed island. This process enhanced not only surrounding ocean turbidity in surface waters very close to the newly formed island, as evidenced by satellite images^14^, but also likely the DFe concentrations observed in surface waters (0.80 nM in 0–30 m) around Tonga (Fig. 1B, St 10–11–12). Nevertheless, below the bottom of the mixed layer, the high enrichment in DFe observed all the way down to 500 m (Fig. 1B) could not be explained by that eruption that likely affected mostly locally surface concentrations. As a result, other sources have been explored.

### Hydrothermal source of Fe

**Figure 2:**
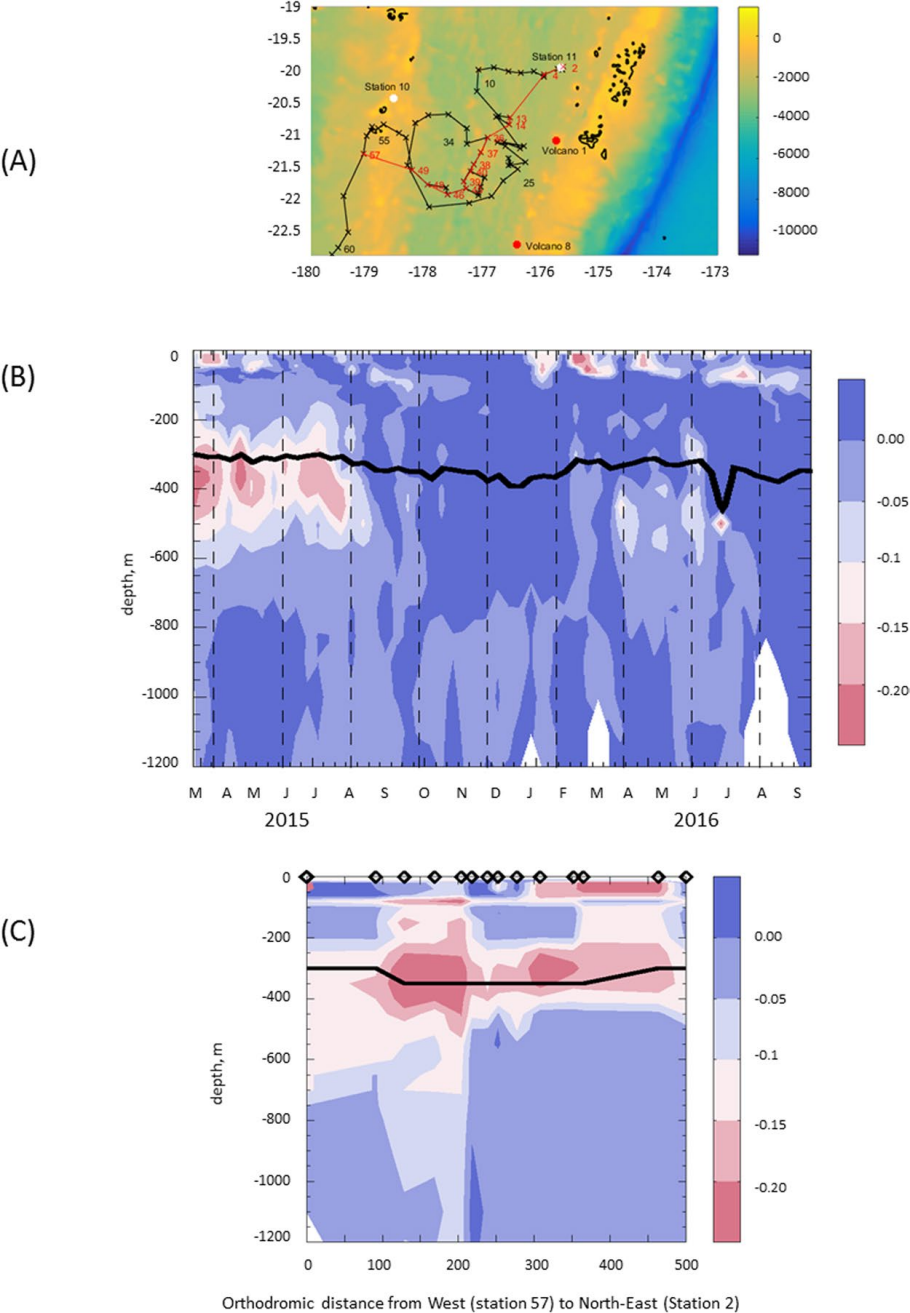
(**A**) Journey of the Argo Float (WMO Id #6901663) (in black) starting at profile 1 performed on March 13, 2015. A non-looping transect (red line between specific stations) is considered in (**C**) to get a spatial mapping, in the float area, away and along the volcanoes. Matlab, 8.6.0.267246 (R2015b), URL link: https://fr.mathworks.com/products/matlab.html was used to generate this map. (**B**) Argo Salinity anomalies along the Argo float journey (anomalies were calculated by subtracting for each profile, a mean annual profile calculated from April 2015 to March 2016), the isopycne 26.0 kg/m^3^ is represented by the black line. (**C**) Impact of a low salinity (5 PSU) source located at Volcano 8 (22°50′S, 176°25′W) on salinity: ocean simulation covering January 2014 to May 2015 with a 300 °C source emitting continuously for 2 months (January 2015 and February 2015) (see Methods) on the red section as in (**A**). The graph represents the salinity differences between the simulation with the source and without. Diamonds on the X-axis in (**C**) represent the position of the ARGO profiles along that section; the x-axis being the orthodromic distance in kilometers (0 = profile 57 to 500 = profile 2).

High DFe concentrations (up to 66 nM) were measured at five profiles at almost all depths between St 7 and St 11, extending zonally over 10 degrees (174 E–175 W longitude) (Fig. 1A). High DFe concentrations (up to 2300 nM) were previously detected in the water column of a shallow volcanic center (∼35 km^2^ at depth above 1600 m; Monowai volcanic center at the northern end of the Kermadec arc^19^), that were attributed to three major hydrothermal fluid effluents linked to the caldera and cone^19^. Micromolar DFe concentrations were also found at similar depths during submarine eruptions in the Extensional NE Lau Basin^20^. These previous studies argue that shallow arc volcano chains, including the Tonga arc of this study located north of the Kermadec arc, may lead to substantial submarine DFe enrichments. A multibeam bathymetric survey along the 650 km-long Tonga arc report the occurrence of 27 submarine volcanoes^21^, nine of them exhibiting volcanic calderas and cones, such as Volcano 1 and 8 (Fig. 2A), which are the closest to our study area. These volcanoes are associated with typical *in situ* hydrothermally-related chemical anomalies in the overlying water column, namely, elevated Fe concentrations^21–25^. Hence, we suspect that the active Volcanos 1 and/or 8 can explain the high DFe concentrations we observed during our cruise (Fig. 1B).

Direct observations of these volcanoes through prior Remotely Operated Vehicle dives revealed the explosive and pulsating nature of the volcanic activity in the WTSP, as evidenced by explosion craters, scoria cones and ash blanket on the caldera floor at ∼500 m^23–25^. In this geodynamic setting, hydrothermal activity displays two different but distinct contributions issued from water-rock interaction, most commonly known as “black smokers” and magmatic-gases due to volcanic activity^26^. The magmatic-hydrothermal system such as the one identified at the Hinepuia volcanic center (Kermadec arc, 26°23′S and 177°16′W), discharges gas-rich (liquid elemental sulfur and probable CO_2_ gas bubbles) but extremely low salinity fluid (∼5 Practical Salinity Unit, PSU) due to subcritical boiling and phase separation^26^. Meanwhile, water-rock hydrothermal systems commonly found at mid-ocean ridges may experience brief (a few days) explosive volcanic events, leading to megaplume formation and dispersion within the water column, i.e., the Juan de Fuca Ridge (North Pacific)^27–29^. In both cases, any submarine volcanic explosion will provoke emission and dispersion of volcanic material (i.e. particles, gases, fluids), which will immediately disrupt the chemical and physical signature of the overlying water column. Thus, it appears that “hydrothermalism” can vary in emission fluxes, duration of the venting (age of the plumes) and chemical characteristics. Whether of magmatic or water-rock hydrothermal origin, the “hydrothermalism” corresponds to a submarine input, a terminology that we have chosen to use for our study.

### Observed salinity anomalies as a proxy for passive tracer of submarine input

Conservative hydrothermal tracers, such as ^3^He, were not measured during the OUTPACE campaign, nor manganese, which is also highly enriched in hydrothermal fluids compared to ambient deep ocean concentrations^30^. Over the last 20 years, deep-sea hydrothermal exploration in the world’s oceans has been facilitated by the use of Autonomous Underwater Vehicles (AUV) equipped with Conductivity Temperature Depth (CTD) instruments coupled to *in situ* optical devices to detect salinity, temperature, optical back-scatter or Eh anomalies associated with hydrothermal plumes^31^. Similarly, autonomous platforms such as Argo floats have been used by physicists to characterize water masses. We took advantage of the *in situ* hydrological characteristics of the water column recorded by an Argo float deployed during the cruise just after Station 11 in March 2015, to test whether the measured high DFe may be related to hydrothermal activity based on the evidence that magmatic-hydrothermal system discharges low salinity fluids^26^. The float remained in the area for more than eighteen months (Fig. 2A) since its deployment and provided vertical profiles of temperature and salinity every 10 days (a total of 57 profiles). Low salinity anomalies were observed (Fig. 2B), associated with temperature anomalies compensating these fresh salinity anomalies and still resulting in a stable water mass. The dynamics of the heat exchanges of the plume is probably complex at this distance from the source and, as salinity is slightly more conservative, it constitutes a better passive tracer than temperature and was thus chosen in our study.

At depths of 200–500 m (i.e., within the main thermocline), negative salinity anomalies up to −0.2 were observed. The surrounding waters represent a mixture of Pacific Equatorial Water flowing from the east and Western South Pacific Central Water flowing from the south. These water masses located in the lower part of the main thermocline (typical range in sigma between 25 and 26.8 kg. m^−3^) are characterized by temperature lower than 14 °C and salinity above 35.1^32^. The observed values represent strong anomalies compared to ambient waters’ values, even including their variability on seasonal to inter-annual timescales. However, due to meso- and submesoscales and potential entrainment conditions within the water column, it remains difficult to isolate, in such anomalies, the exact contribution resulting from hydrothermal activity. These anomalies are located right in the sector where the high DFe concentrations were measured (Fig. 1). Despite the lack of temporal synopticity with the Fe data collected during the cruise, the Argo data displayed recurrent S anomalies for several months in the area where it stayed since its deployment. Mesoscale activity such as eddies can transport water masses with different characteristics throughout the WTSP^33^. Nonetheless, in the specific depth range of this area, no other typical water masses have S values as low as the ones measured opportunely by this float. A low S water mass may be linked to magmatic and/or water-rock hydrothermal activity, as deep-sea hydrothermal fluids collected immediately after a volcanic eruption at mid-ocean ridge axis^34^ or at magmatic-hydrothermal system such as the one at Hinepuia^26^, which exhibited unusual extremely low salinity (below 50 mM of chlorine concentration, i.e. salinity <5). This is due to enhanced degrees of phase separation and high H_2_, H_2_S and CO_2_ magmatic degassing^30,35^. Interestingly, Fe solubility during water-rock interactions is significantly increased in low-salinity vapor-dominated hydrothermal fluids^34^, implying enhanced DFe flux to the water column.

### Modeled salinity anomalies

The possibility that a continuous or intermittent submarine source located at Volcano 1 or Volcano 8 (Fig. 2A) can indeed impact the surface ocean hydrological characteristics is considered by means of an ocean general circulation model (OGCM^36^). The strategy chosen here is not to detail mechanisms of hydrothermal fluid injection into the ocean, which require a thorough knowledge of the source characteristics^37^. In the absence of such data, our strategy was to explore the sensitivity of the model response to submarine emissions in several simulations (Method, Table 1) based on documented durations, flux, salinity and temperature of the emitted fluid over the time-scale defined by the Argo float deployment, (i.e. a few months). Using a relatively idealized 1-D framework, Speer and Rona (1989) explored the adjustment of a high temperature hydrothermal plume in the Pacific venting at 2200 m. They showed that the high temperature hydrothermal fluid (350 °C) achieved neutral buoyancy in the water column at ~200 m above seafloor indicating that, in their case, the maximum vertical course of the fluid emitted at 350 °C was ~200 m. Our 3-D OGCM experiments with state-of-the art 3-D mechanisms at play in the open ocean uses a similar source temperature emission with a very low salinity at an injection depth much shallower (between 450 and 500 mbsl). This permits scientists to explore the fate of the fluid anomalies in the vertical and horizontal directions as realistically as possible. The mean state of our reference model simulation (Methods) compares favorably with the observations in terms of salinity (Supplementary Fig. 2) and temperature (not shown). Simulations taking into account a flux of 500 l s^−1^^38^ of a 230 °C fluid whose salinity is 20 PSU over a 6-month duration, showed a maximal salinity anomaly ~0.15 weaker than the one observed,with a shorter-duration (2 months) emission. Indeed increasing 20 times the flux (similar to that calculated during a megaplume event^28^), but on a shorter duration, the plume anomaly and intensity increased, and there was good agreement with the observations. When the temperature was increased to 300 °C and the salinity decreased to 5 PSU (Fig. 2C and Supplementary Fig. 3), the model outputs were closer to the observations with the same flux (10 000 l s^−1^) and duration (2 months). An intermittent event of high magnitude is thus likely to have caused the observed anomaly. The numerous earthquakes recorded in this area during our study period (Supplementary Fig. 5) also support this hypothesis.

**Table 1:** Parameters chosen for the submarine emissions in the simulations based on source flux^38,63,64^, source temperature^65^, and source salinity^66^.

*Source location*	volcano 1	21°09′S 175°45′W	source depth, m 450
	volcano 8	22°50′S 176°25′W	source depth, m 500
*Source Flux, L.s-1*	500–10000 l.s^−1^		
*Source temp, °C*	230–300		
*Source Salinity, PSU*	5–15–20		
*Emission duration, months*	2 months, 6 months		

### Spatial extent of the plume

A low salinity source emitting at 300 °C for two months, led to a first order negative salinity anomaly of ~0.2 at approximately 200 km away from the volcanic source (Supplementary Fig. 3), which is consistent with our observations (Fig. 2B). Remarkably, the modeled submarine source at the caldera seafloor is mixed by convection to a density level consistent with the observations. Modeled salinity anomalies are concentrated within the 200–400 m layer and are also visible in the photic layer (0–90 m). They have a tendency to reach a maximum at approximately 300 m in the meridional section and over several degrees of longitude (Supplementary Fig. 3), at locations in agreement with those observed. Interestingly, the depth reached by these maximum anomalies (~300 m) is 150 m above the depth of the venting, a vertical distance of plume rise compatible with that found more theoretically in previous research^39^. In our case, the model allows to us to follow the anomalies spatially (Supplementary Fig. 3a–d) as the ocean currents advect and diffuse the initial anomalies of Volcano 8. The model also shows how eddies strongly modulate the west/north westward general movement of the initial anomaly. Water mass movements and entrainment of deep waters in upper layers can occur over steep or highly variable topography. Nonetheless, here, this effect has been ruled out since the model exhibits salinity anomalies only when strong and intermittent emissions of a low salinity fluid at Volcano 1 or 8 are considered. The presence of significant salinity anomalies is also detected in Argo atlas from the ISAS13 product^40^ with similar properties at depth (and along the same isopycnal surfaces) and the same timing between 2014 and 2015 (Supplementary Fig. 4). The submarine plume diffuses and gets advected. Within a few months, the modeled salinity anomaly propagates over a large area west and north-west of the source (Supplementary Fig. 3), consistent with the main predominant westward currents associated with the subtropical gyre, and modulated by mesoscale activity.

## Discussion

Results strongly support the remote and crucial role of a shallow, intermittent and strong submarine source located in the Tonga arc, in shaping the spatial and temporal DFe field observed during OUTPACE (Fig. 1B). Interestingly, the submarine Fe source directly impacted the DFe concentrations in the photic layer. These concentrations reached 3.8 nM on average between stations Stations 7 and 11. Although it was recently hypothesized^41^ that Fe from hydrothermal inputs from shallow submarine island arc calderas systems (such as the Tonga–Kermadec arc) could reach the productive zone of the sea surface, this is rarely observed in the ocean as most mid-ocean hydrothermal Fe sources are usually located deeper (∼3000 m, e.g.^42^).

As depicted by the spatial propagation of the salinity anomaly (Supplementary Fig. 3), high mesoscale activity in the region such as eddies^33^ can transport, disperse and maintain high DFe at the WTSP scale. This statement implies that most of DFe from shallow hydrothermal submarine source is not lost from solution during transport, and mixes almost conservatively with ambient waters. While there is recent evidence for such behavior for DFe from hydrothermal venting in deep-ocean ridges on long spatial scales^42,43^, this is the first time that this is assumed for shallow hydrothermal inputs. The maintaining of DFe in solution depends on stabilization mechanisms such as the complexation by organic ligands. Hydrothermal sources release high quantities of organic compounds, enabling the stabilization and preservation of metals such as Fe as organic-binding complexes in the water column^44,45^. Although we only measured total DFe, previous modeling experiments have shown that such ligands are needed to explain the persistence of DFe far from the source emissions in the deep ocean^42^. Whether similar ligands and/or ligands in the surface ocean seawater can explain the persistence of DFe in shallow environment is unknown.

Farther east of Tonga, in the large South Pacific Gyre (SPG), the seafloor goes down to 5000 m, preventing any Fe fertilization of the productive layer from below. Indeed, the ferricline is likely deeper than the pycnocline (found above 400 m^46^) as the concentrations are low and homogeneous throughout the entire 0- to 500-m profile (Fig. 1). Within the gyre at the same latitude, Fitzsimmons and coauthors (2016)^47^ calculated vertical diffusive DFe fluxes through the ferricline of 1.5 to 2.8 µmol DFe.m^−2^ yr^−1^ and showed that these fluxes were three orders of magnitude lower than horizontal diffusive fluxes. In the SPG (170–90 W), atmospheric deposition of Fe is among the lowest in the world’s oceans^48^. DFe concentrations are in the range of 0.1–0.3 nM in the water column^49,50^ (and this study). This likely prevents N_2_ fixation from occurring, as rates were close to detection limits in this region during the OUTPACE cruise^51^, consistent with former studies^52^. The quasi-absence of N_2_ fixation likely explains why dissolved inorganic phosphorus (DIP) is not consumed, accumulates in surface waters in the absence of nitrate, and why the system turns to ultra-oligotrophic conditions^53^. In contrast, west of 170 W, the WTSP waters represent a high DFe, high SST (>28 °C during austral summer conditions) ecosystem receiving DIP-enriched waters flowing from the east through the South Equatorial Current (SEC). N_2_ fixation rates measured during OUTPACE in this region are among the highest reported in the global ocean (average 631 ± 286 µmol N m^−2^ d^−1^^51^ compared to the common range of 10–100 µmol N m^−2^ d^−1^ reported for the global tropical ocean^54^. Our study reveals that a shallow submarine Fe source in the region of the Tonga arc fertilizes photic waters of the WTSP. This is of the utmost importance as it has potentially strong impacts on ecosystem functioning as N_2_ fixation fuels nearly all new primary production and organic matter export in the WTSP during austral summer conditions^55^. Indeed, between this shallow submarine Fe source and the DIP-enriched waters of the SEC, conditions in the WTSP are likely ideal for diazotrophs to bloom extensively and probably explains the hotspot of N_2_ fixation^1^.

Future investigations are required to quantify this Fe flux from below, study the scavenging/mixing fate of hydrothermal plumes in the water column at the local and regional scales, characterize submarine sources (hydrothermal vs. volcanic), characterize metal vs. ligand sources, and quantify the biogeochemical impact of shallow submarine hydrothermal sources on biological pump processes such as primary production, nitrogen fixation, and export production.

## Methods

### DFe measurements

A total of 186 water samples from 16 vertical profiles (0–500 m) were sampled using a Titanium Rosette mounted with 24 Teflon-coated 12 L GoFlos and operated along a Kevlar cable. GoFlos were transported inside a clean container, and samples were filtered directly from the GoFlos through 0.2-µm cartridges (Sartorius Sartrobran-P-capsule with a 0.45-μm prefilter and a 0.2-μm final filter), following trace metal clean protocols described previously^50^. DFe concentrations were measured by flow injection with online preconcentration and chemiluminescence detection using the exact protocol, instrument, and analytical parameters as described previously^50^. Some of the samples were analyzed at sea, and the remaining acidified samples were analyzed at LOV. The reliability of the method was monitored by analyzing the D1 SAFe seawater standard^56^, and an internal acidified seawater standard was measured every day in order to monitor the stability of the analysis. The same methodology was also used to analyze DFe from dissolution experiments. Analyses of SAFe D1 standard^56^ for DFe averaged 0.62 ± 0.06 nmol/kg (n = 9), which agrees well with the consensus value of 0.67 ± 0.04 nmol/kg as of May 2013 (http://www.geotraces.org/science/intercalibration/322-standards-and-reference-materials). Considering the wide range of [DFe], calibration curves and pre-concentration times were adapted in order to measure DFe concentrations within the calibration curves. Preconcentration was 120 s for most of the samples and down to 10 s for the highest [DFe]. All the measured concentrations are reported in Supplementary Table 2.

### Aerosol sampling and measurements

Ten aerosol samples were collected during the cruise transect (Supplementary Fig. 1) for total Fe concentrations measurements and to quantify their potential DFe release in seawater. Aerosol samples were collected using a sampling device designed to avoid ship contamination. This sampling device (fully described in^48^) was installed at the look-out post in the front on the ship (10 m above sea level). Briefly, the device was able to collect four samples simultaneously at ~20 L. min^−1^, (onto polycarbonate, 47-mm diameter, 0.45-μm porosity previously acid-cleaned with a 2% solution of HCl (Merck, Ultrapur, Germany) and thoroughly rinsed with ultra-pure water and dried under a laminar flow bench and stored in acid-cleaned Petri dishes). The total amount of air pumped through each filter was recorded using volumetric counters. Wind direction and speed were measured continuously close to the sampling device using a wind vane coupled to an anemometer. Depending on the wind conditions, the device operated either in ‘sampling’ mode or ‘protection’ mode. Sampling mode (air pumped through the filters) was activated only if wind was oriented at an open 90-degree angle upwind at a speed higher than 2 m s^−1^. Protection mode (no air pumped, device closure, and filters protected) was activated if wind conditions could generate sample contamination. A total of 10 × 4 samples (Aero1 to Aero11) were collected and used for (1) total iron analysis (SFX) and (2) dissolution experiments.

Aerosols total iron concentrations. The total iron concentrations were obtained by wavelength dispersive X-ray fluorescence (WD-XRF) for samples collected during the cruise, using a PW-2404 spectrometer by PANalytical following exactly the same protocol as described by^57^.

### Aerosols dissolution experiments

Dissolution experiments were conducted on aerosol samples. Using acid-cleaned Sartorius filtration units (volume 0.250 L) and seawater collected at 4 different stations along the transect, the samples were subjected to two contact times with filtered seawater: the first contact was at one minute, and the second contact was at 24 hours. DFe was measured in the filtrates by FIA (see above).

### Argo Float data

Information on the Arvor float trajectory and data can be found at: http://www.coriolis.eu.org/Data-Products/Data-Delivery/Argo-floats-by-WMO-number, entering its float number: 6901663. Salinity are reported with a resolution on the order of 0.01 pss-78 (practical salinity scale, according to the 1978 practical salinity scale).

### Simulation of salinity anomaly

The OUTPACE and float data, despite being quite informative, were not meant for a study oriented towards submarine sources. Hence, we decided that the best strategy was to rely on the results from an OGCM simulation. We thus used the Regional Oceanic Modeling System (ROMS^58^ to explore the impact of volcanic sources at the ocean floor on salinity and to compare our simulations with the numerous *in situ* observations allowed by the Argo Float (see previous section). As well as this reference simulation, we performed several simulations with submarine sources located either at Volcano 1 or Volcano 8 and characterized by different physico-chemical parameters (Table 1). These sources are modeled as rivers discharging from the caldera seafloor. Our 3-D model framework was chosen over a 1-D diffusive model^39^ because our model not only includes the vertical diffusion processes of ^39^ but also permits to follow the fate of the water masses 3-dimensionally as obviously observed off the hydrothermal source (Supplementary Fig. 3).

Our regional configuration is based on that of ^36^. The model domain is [165°E–167.5°W/23.5°S–15°S], and the spatial resolution is 1/12° in latitude and longitude. It has 41 terrain-following vertical levels, leading to a vertical resolution of 2 to 5 -m within 50 m of the ocean surface and 10 to 20 -m in the thermocline. Open boundary conditions^59^ are specified using the mercator ocean reanalysis (http://marine.copernicus.eu/services-portfolio/access-to-products/?option=com_csw&view=details&product_id=GLOBAL_ANALYSIS_FORECAST_PHY_001_024). The reference simulation spans January 2014 to May 2015, and the model initial state is taken from that reanalysis. The model time step is 1/3 hours. At six-hour intervals, heat, fresh and momentum fluxes used to force the reference model simulation were calculated using^60^ bulk formulae with NCEP2^61^ surface atmospheric inputs for the heat and freshwater fluxes, but the ERA interim^62^ was used for the wind stress to maintain compatibility with the ocean re-analysis at the boundaries.

## Electronic supplementary material

Supplementary Information
