# Supplementary material for: Iron from a submarine source impacts the productive layer of the Western Tropical South Pacific (WTSP)
Source: Sci Rep. 2018 Jun 13;8:9075. doi: 10.1038/s41598-018-27407-z (PMC5998060; doi:10.1038/s41598-018-27407-z)
Supplement: Supplementary file 1 — Supplementary Information [file 41598_2018_27407_MOESM1_ESM.docx]

Supplementary Information

**Iron from a submarine source impacts the productive layer of the Western Tropical South Pacific (WTSP)**

**Authors:** C. Guieu^1,2^, S. Bonnet^3^, A. Petrenko^4^, C. Menkes^5^, V. Chavagnac^6^, K. Desboeufs^7^, C. Maes^8^, T. Moutin^4^

^1^ Sorbonne Universités, UPMC Univ Paris 06, INSU-CNRS, Laboratoire d'Océanographie de Villefranche, 181 Chemin du Lazaret, 06230 Villefranche-sur-mer, France

^2^ Center for Prototype Climate Modeling, New York University Abu Dhabi, P.O. Box 129188, Abu Dhabi, United Arab Emirates

^3^Aix Marseille Université, Toulon Université, CNRS, Institut de Recherche pour le Développement (IRD), Observatoire des Sciences de l'Univers Pythéas, Mediterranean Institute of Oceanography (MIO), Unité Mixte 110, 98848 Noumea, New Caledonia

^4^Aix Marseille Université, CNRS/Institut National des Sciences de l’Univers, Université de Toulon, Institut de Recherche pour le Développement, Observatoire des Sciences de l'Univers Pythéas, Mediterranean Institute of Oceanography (MIO), Unité Mixte 110, 13288 Marseille, France

^5^ LOCEAN (Université Sorbonne - Pierre et Marie Curie, IRD/CNRS/MNHN), IRD, BP A5,

98848 Nouméa Cedex, New Caledonia

^6^ Géosciences Environnement Toulouse, GET UMR5563, CNRS/UPS/IRD/CNES, Observatoire Midi-Pyrénées, 14 Avenue Edouard Belin, 31400 Toulouse, France

^7^ Laboratoire Interuniversitaire des Systèmes Atmosphériques (LISA), IPSL, UMR CNRS 7583, Université Paris Est Créteil (UPEC), Université Paris Diderot (UPD), Créteil, France

8 IRD/LOPS, IFREMER, CNRS, IUEM, University of Brest, Brest, France, orcid.org/0000-0001-6532-7141

correspondence to:  guieu@obs-vlfr.fr

**This PDF file includes:**

1. Supplementary Figures 1 to 5
2. Supplementary Tables 1 to 2
3. **Supplementary Figures 1 to 5**


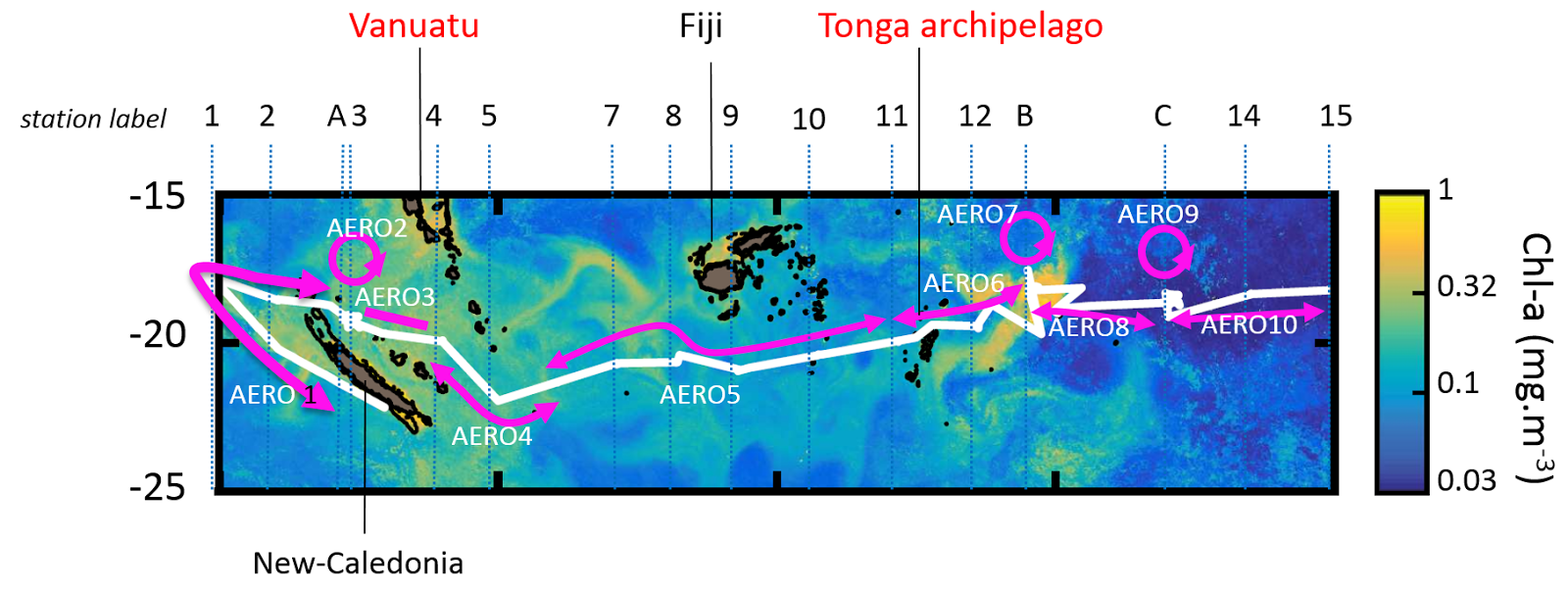


**Supplementary Figure 1. Segments of transect corresponding to the different aerosols sampled during the OUTPACE cruise. (The ocean color satellite products are produced by CLS. Figure courtesy of A. De Verneil)**


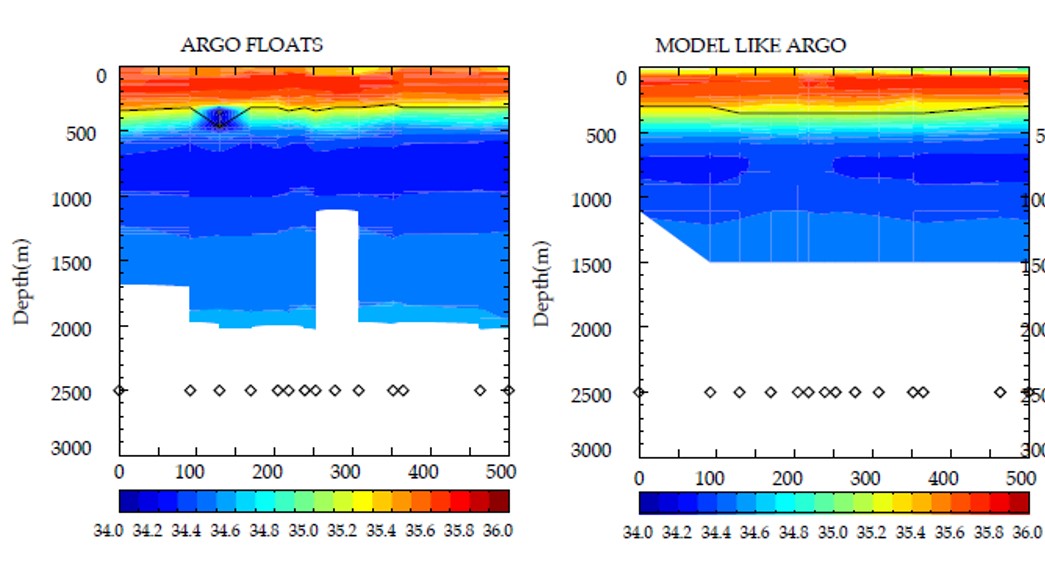


**Supplementary Figure 2. Vertical sections of salinity for the ARGO float (along the red segment in Fig. 2A) (left) and reference model outputs averaged for February 2015 to May 2015 (right). Diamonds on the X-axis represent the position of the ARGO profiles along that section; the x-axis being the orthodromic distance in kilometers (0 = profile 57 to 500 = profile 2). The model reproduces the major features and typical range of values observed in that region.**

**(a)
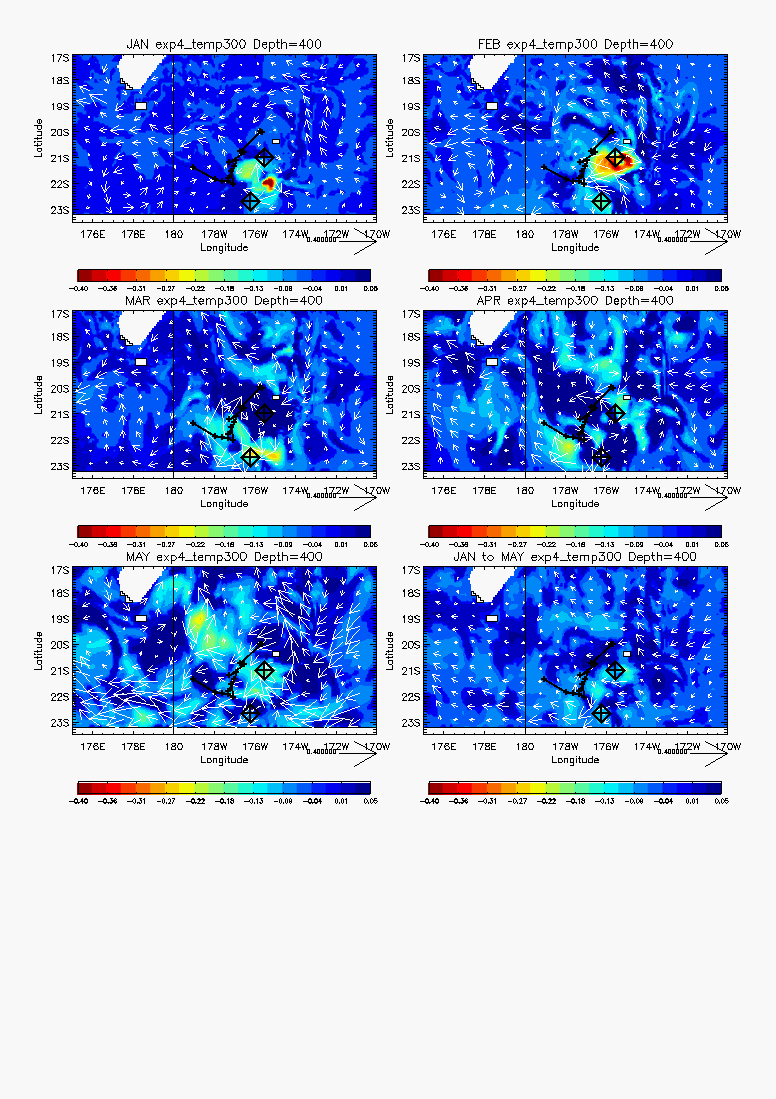
**

**(b)
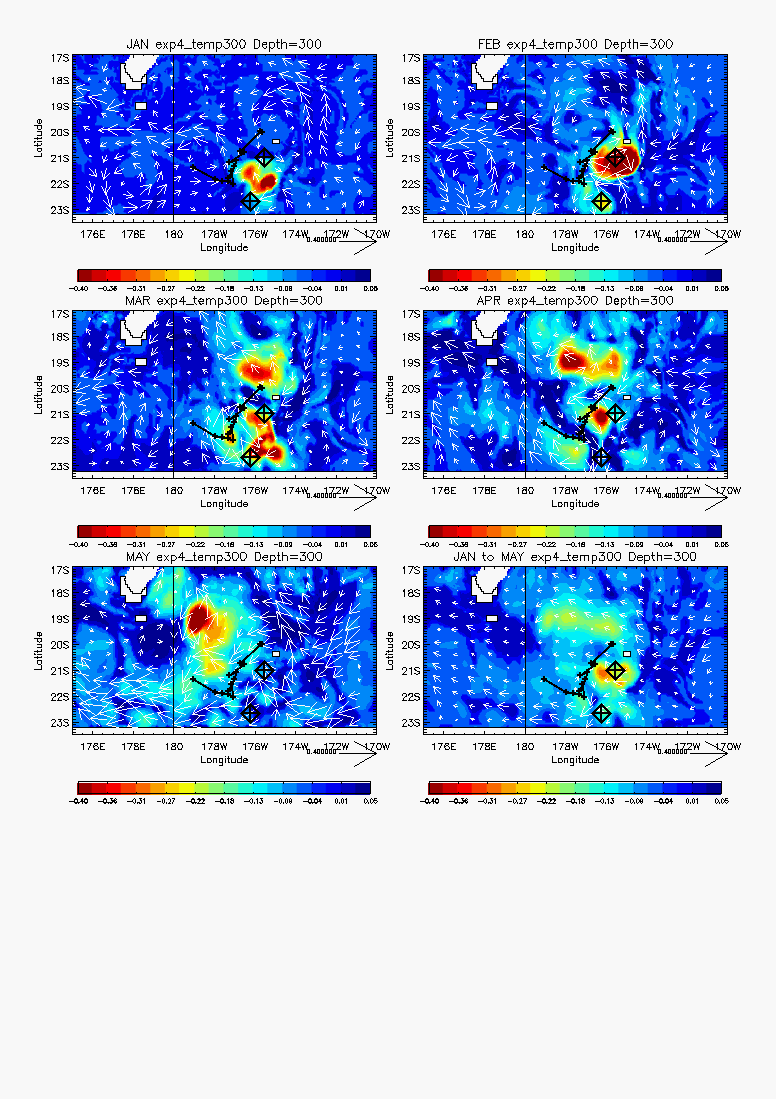
**

**(c)
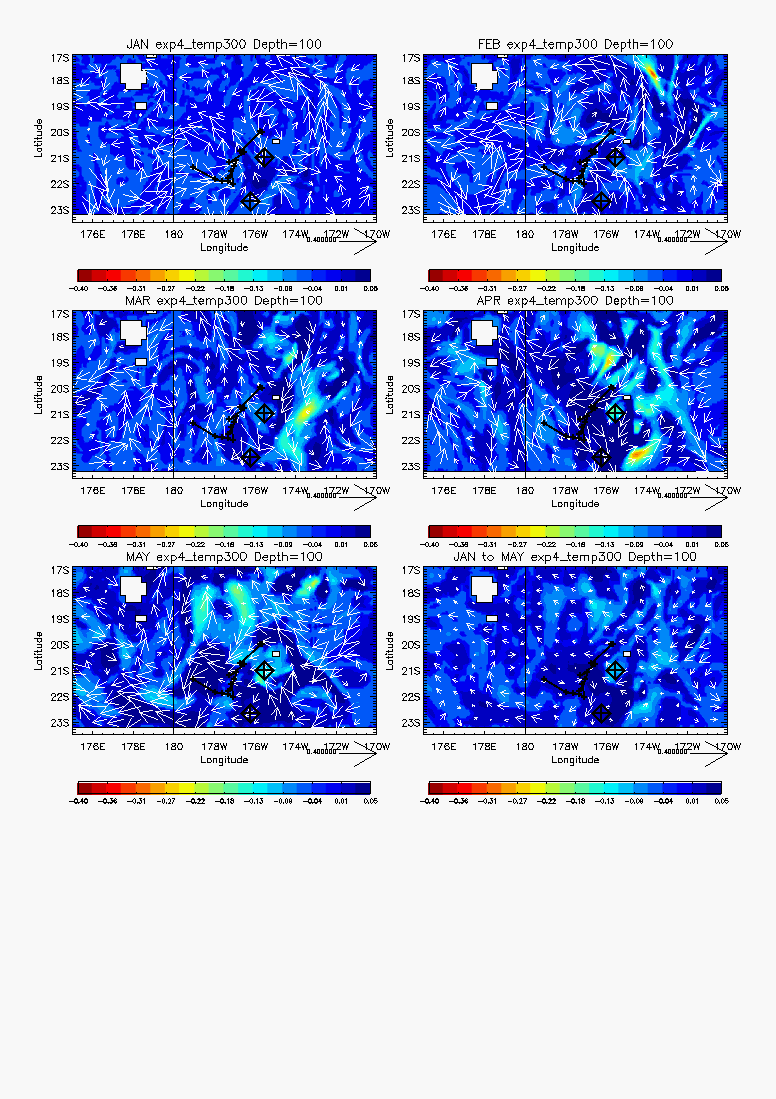
**

**(d)
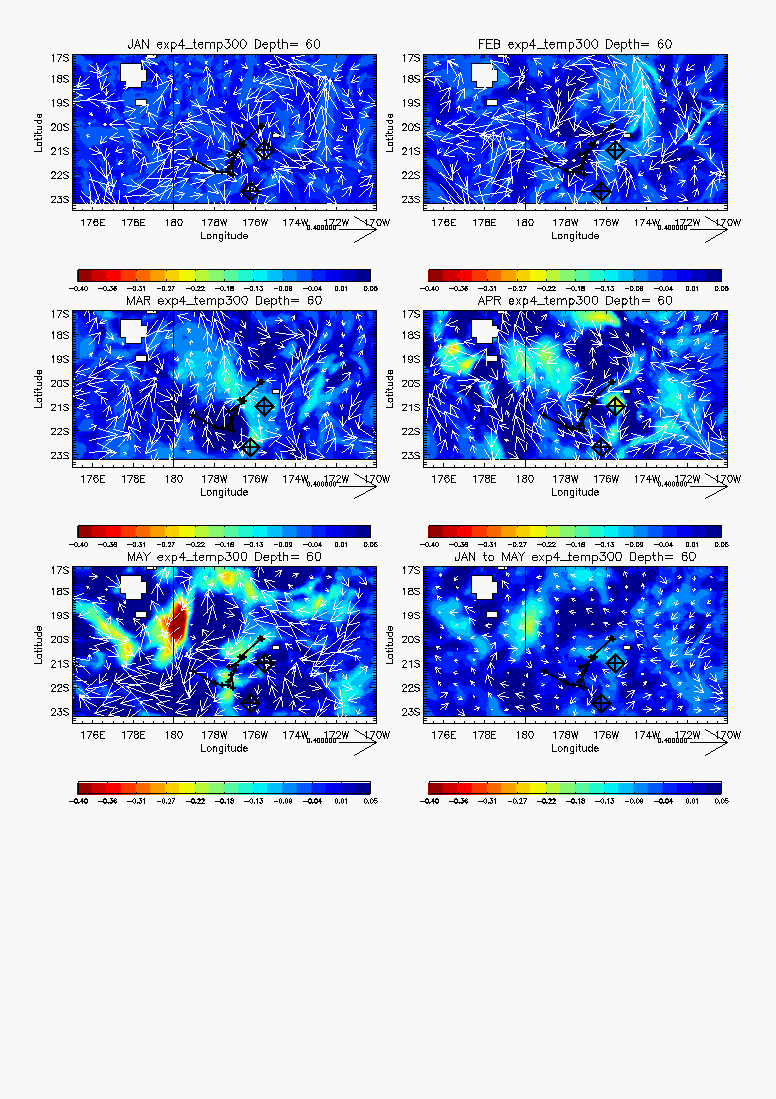
**

**Supplementary Figure 3.  Time mean section of the January 2015 to May 2015 simulations to characterize spatial and temporal plume impacts taking Volcano 8 as a source location that emitted throughout December 2014 and January 2015 (same conditions as in Fig. 2C : salinity of the source = 5, temperature = 300°, duration of the emission = 2 months, flux = 10 000 l.s^-1^). Monthly mean snapshots of the region from January to May 2015 (from left to right and top to bottom), the last panel representing the mean of February to May 2015. On each panel, salinity anomalies (model with volcano 8 plume minus model without) are shaded and horizontal currents are represented as vectors. These snapshots are represented at 400 m (a), 300 m (b), 100 m (c) and 50 m (d). On each panel, the position of the ARGO float section of Fig. 2A is represented by a  black line and the positions of Volcano1 and 8 are noted as crossed diamonds. IDL version 6.4, Mac OSX (Darwin) , (c) 2007 ITT Visual Information Solutions URL link :** [**http://www.harrisgeospatial.com/SoftwareTechnology/IDL.aspx**](http://www.harrisgeospatial.com/SoftwareTechnology/IDL.aspx) **was used to generate the maps.**

**
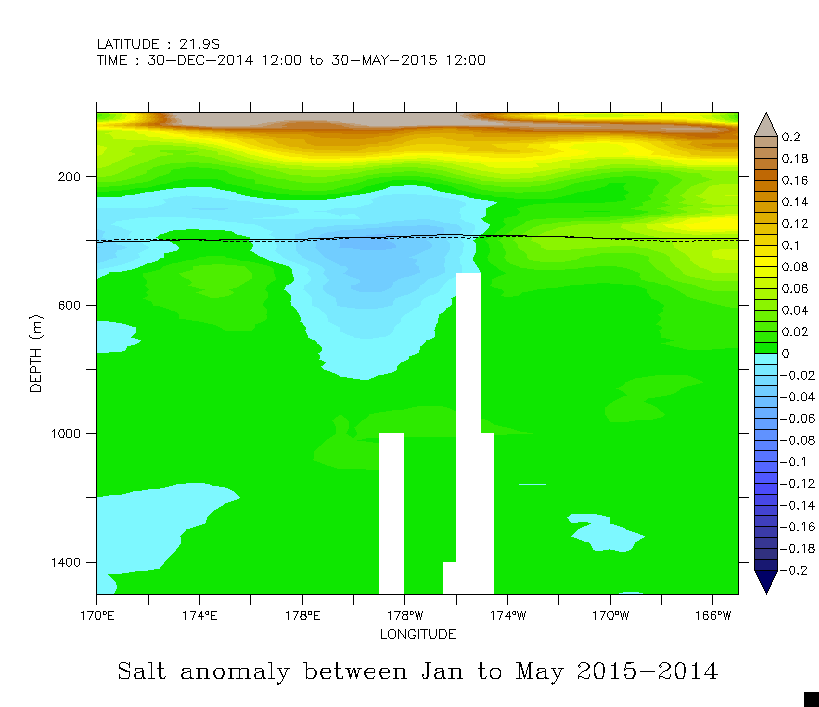
**

**Supplementary Figure 4. Differences of the average salinity section over the Jan-May period between 2015 and 2014 along 22°S from the ISAS13 Argo atlas. The quite similar depth of the 26.5 kg/m3 isopycnal surface for 2015 (thin line) and 2014 (dash line) indicates that this salt anomaly is compensated by a cold anomaly of (0.2°C) during the same period.**

**
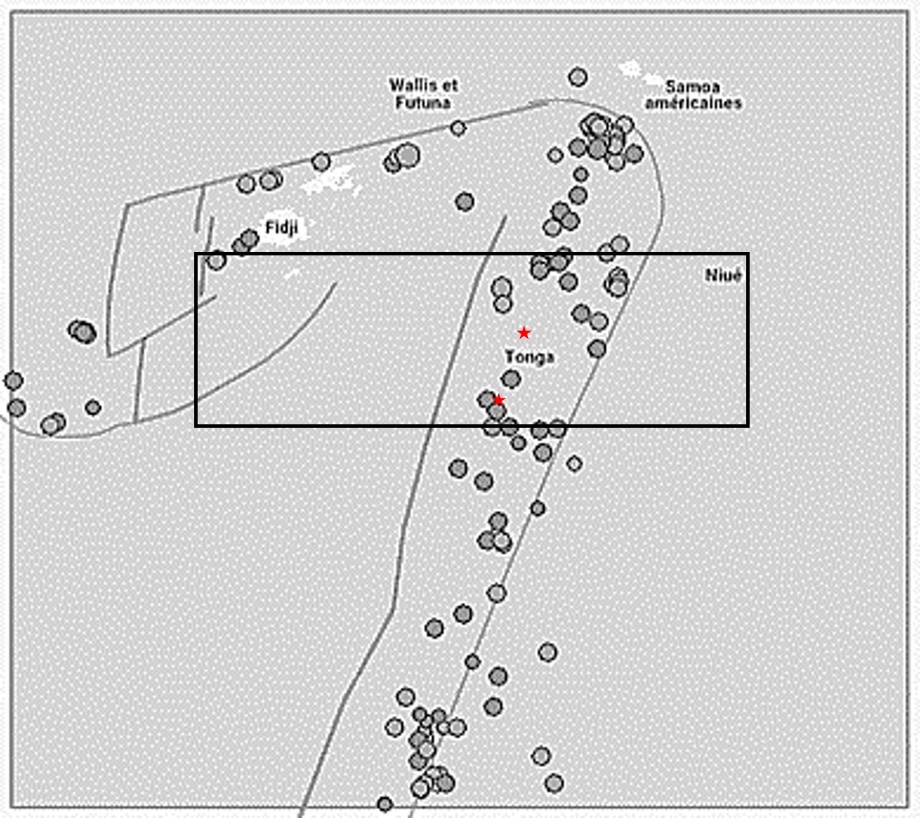
**

**Supplementary Figure 5. Earthquakes (magnitude > 4 and above 100 km) recorded during the period 01/12/2014 to 01/06/2015, in our study area (black box; red stars: volcano 1 and 8). (this map was generated using https://earthquake.usgs.gov/).**

1. **Supplementary Tables 1 to 2**

**Supplementary Table 1. Aerosols results: total iron concentrations and fluxes; soluble iron concentrations and fluxes. The segments where aerosols were sampled are indicated Supplementary Figure 1.**

| **Aerosol samples labels** | **Fe concentrations** | **Total Fe flux** | **DFe concentrations** | **DFe fluxes** | **DFe fluxes** |
| --- | --- | --- | --- | --- | --- |
|  | nM.m-3 | nM.m-2.d-1 | nM.m-3 | nM. m-2. d-1 | mg.m-2.yr-1 |
| **AERO 1** | 1.375 | *1663* | nd |  |  |
| **AERO 2** | 0.041 | *49* | nd |  |  |
| **AERO 3** | 0.013 | *15* | nd |  |  |
| **AERO 4** | 0.064 | *78* | 0.0012 | *1.42* | *0.029* |
| **AERO 5** | nd |  | nd |  |  |
| **AERO 6** | 0.162 | *196* | 0.0027 | *3.25* | *0.066* |
| **AERO 7** | nd |  | 0.0020 | *2.46* | *0.050* |
| **AERO 8** | 0.085 | *102* | 0.0402 | *48.7* | *0.992* |
| **AERO 9** | nd |  | 0.0007 | *0.85* | *0.017* |
| **AERO 10** | 1.96 | *2371* | 0.0104 | *12.6* | *0.256* |

**Supplementary Table 2. Dissolved iron, nM measured between 0-500m during the OUTPACE cruise.**

| Latitude | Longitude | Station | Depth (m) | DFe nM |
| --- | --- | --- | --- | --- |
| -18 | 159.9 | 1 | 500 | 0.85 |
| -18 | 159.9 | 1 | 400 | 0.60 |
| -18 | 159.9 | 1 | 350 | 0.88 |
| -18 | 159.9 | 1 | 300 | 0.59 |
| -18 | 159.9 | 1 | 250 | 0.60 |
| -18 | 159.9 | 1 | 200 | 0.62 |
| -18 | 159.9 | 1 | 150 | 0.53 |
| -18 | 159.9 | 1 | 90 | 0.42 |
| -18 | 159.9 | 1 | 80 | 0.31 |
| -18 | 159.9 | 1 | 50 | 0.50 |
| -18 | 159.9 | 1 | 30 | 0.92 |
| -18 | 159.9 | 1 | 10 | 0.67 |
| -18.6 | 162.1 | 2 | 500 | 0.42 |
| -18.6 | 162.1 | 2 | 400 | 0.36 |
| -18.6 | 162.1 | 2 | 350 | 0.37 |
| -18.6 | 162.1 | 2 | 300 | 0.39 |
| -18.6 | 162.1 | 2 | 250 | 0.56 |
| -18.6 | 162.1 | 2 | 200 | 0.36 |
| -18.6 | 162.1 | 2 | 150 | 0.37 |
| -18.6 | 162.1 | 2 | 100 | 0.26 |
| -18.6 | 162.1 | 2 | 70 | 0.24 |
| -18.6 | 162.1 | 2 | 50 | 0.99 |
| -18.6 | 162.1 | 2 | 30 | 0.41 |
| -18.6 | 162.1 | 2 | 10 | 0.36 |
| -19.2 | 164.7 | LD_A | 500 | 0.50 |
| -19.2 | 164.7 | LD_A | 450 |  |
| -19.2 | 164.7 | LD_A | 350 | 0.50 |
| -19.2 | 164.7 | LD_A | 300 | 0.48 |
| -19.2 | 164.7 | LD_A | 250 | 0.49 |
| -19.2 | 164.7 | LD_A | 200 | 0.51 |
| -19.2 | 164.7 | LD_A | 80 | 0.52 |
| -19.2 | 164.7 | LD_A | 50 | 0.50 |
| -19.2 | 164.7 | LD_A | 30 | 0.85 |
| -19 | 165 | 3 | 500 | 0.43 |
| -19 | 165 | 3 | 400 | 0.31 |
| -19 | 165 | 3 | 350 | 0.58 |
| -19 | 165 | 3 | 300 | 0.44 |
| -19 | 165 | 3 | 250 | 0.42 |
| -19 | 165 | 3 | 200 | 0.62 |
| -19 | 165 | 3 | 150 | 0.21 |
| -19 | 165 | 3 | 100 | 0.85 |
| -19 | 165 | 3 | 70 | 0.37 |
| -19 | 165 | 3 | 50 | 0.48 |
| -19 | 165 | 3 | 30 | 0.32 |
| -19 | 165 | 3 | 10 | 0.38 |
| -20 | 168 | 4 | 500 | 0.60 |
| -20 | 168 | 4 | 400 | 0.85 |
| -20 | 168 | 4 | 350 | 0.67 |
| -20 | 168 | 4 | 300 | 0.82 |
| -20 | 168 | 4 | 250 | 0.63 |
| -20 | 168 | 4 | 200 | 0.56 |
| -20 | 168 | 4 | 150 | 0.78 |
| -20 | 168 | 4 | 100 | 0.41 |
| -20 | 168 | 4 | 70 | 0.54 |
| -20 | 168 | 4 | 50 | 1.16 |
| -20 | 168 | 4 | 30 | 0.55 |
| -20 | 168 | 4 | 10 | 0.69 |
| -22 | 170 | 5 | 500 | 0.44 |
| -22 | 170 | 5 | 400 | 0.62 |
| -22 | 170 | 5 | 350 | 0.35 |
| -22 | 170 | 5 | 300 | 1.17 |
| -22 | 170 | 5 | 250 | 1.25 |
| -22 | 170 | 5 | 200 | 0.26 |
| -22 | 170 | 5 | 150 | 0.24 |
| -22 | 170 | 5 | 100 | 1.01 |
| -22 | 170 | 5 | 70 | 0.50 |
| -22 | 170 | 5 | 50 | 0.53 |
| -22 | 170 | 5 | 30 | 0.61 |
| -22 | 170 | 5 | 10 | 0.44 |
| -20.73 | 174.27 | 7 | 500 | 0.32 |
| -20.73 | 174.27 | 7 | 400 | 0.44 |
| -20.73 | 174.27 | 7 | 350 | 0.44 |
| -20.73 | 174.27 | 7 | 300 | 0.43 |
| -20.73 | 174.27 | 7 | 250 | 0.44 |
| -20.73 | 174.27 | 7 | 200 | 0.46 |
| -20.73 | 174.27 | 7 | 150 | 0.39 |
| -20.73 | 174.27 | 7 | 100 | 0.21 |
| -20.73 | 174.27 | 7 | 70 | 2.88 |
| -20.73 | 174.27 | 7 | 50 | 0.25 |
| -20.73 | 174.27 | 7 | 30 | 1.22 |
| -20.73 | 174.27 | 7 | 10 | 0.21 |
| -20.7 | 176.4 | 8 | 500 | 0.47 |
| -20.7 | 176.4 | 8 | 400 | 0.41 |
| -20.7 | 176.4 | 8 | 350 | 0.42 |
| -20.7 | 176.4 | 8 | 300 | 1.38 |
| -20.7 | 176.4 | 8 | 250 | 0.42 |
| -20.7 | 176.4 | 8 | 200 | 0.33 |
| -20.7 | 176.4 | 8 | 150 | 0.26 |
| -20.7 | 176.4 | 8 | 120 | 0.31 |
| -20.7 | 176.4 | 8 | 70 | 0.22 |
| -20.7 | 176.4 | 8 | 50 | 0.45 |
| -20.7 | 176.4 | 8 | 30 | 1.35 |
| -20.7 | 176.4 | 8 | 10 | 0.38 |
| -21 | 178.6 | 9 | 500 | 0.39 |
| -21 | 178.6 | 9 | 398 | 1.90 |
| -21 | 178.6 | 9 | 350 | 0.42 |
| -21 | 178.6 | 9 | 300 | 1.60 |
| -21 | 178.6 | 9 | 250 | 5.50 |
| -21 | 178.6 | 9 | 200 | 66.20 |
| -21 | 178.6 | 9 | 150 | 12.10 |
| -21 | 178.6 | 9 | 100 | 63.00 |
| -21 | 178.6 | 9 | 70 | 0.76 |
| -21 | 178.6 | 9 | 50 | 0.25 |
| -21 | 178.6 | 9 | 30 | 0.22 |
| -21 | 178.6 | 9 | 10 | 0.22 |
| -20.5 | 181.5 | 10 | 500 | 2.15 |
| -20.5 | 181.5 | 10 | 400 | 1.55 |
| -20.5 | 181.5 | 10 | 350 | 7.80 |
| -20.5 | 181.5 | 10 | 300 | 0.87 |
| -20.5 | 181.5 | 10 | 250 | 4.80 |
| -20.5 | 181.5 | 10 | 200 | 1.17 |
| -20.5 | 181.5 | 10 | 150 | 0.16 |
| -20.5 | 181.5 | 10 | 120 | 5.17 |
| -20.5 | 181.5 | 10 | 70 | 11.32 |
| -20.5 | 181.5 | 10 | 50 | 0.94 |
| -20.5 | 181.5 | 10 | 30 | 0.43 |
| -20.5 | 181.5 | 10 | 10 | 0.97 |
| -19.98 | 184.33 | 11 | 500 | 0.42 |
| -19.98 | 184.33 | 11 | 400 | 0.47 |
| -19.98 | 184.33 | 11 | 350 | 0.51 |
| -19.98 | 184.33 | 11 | 300 | 1.08 |
| -19.98 | 184.33 | 11 | 250 | 0.63 |
| -19.98 | 184.33 | 11 | 200 | 0.59 |
| -19.98 | 184.33 | 11 | 150 | 0.64 |
| -19.98 | 184.33 | 11 | 100 | 0.72 |
| -19.98 | 184.33 | 11 | 70 | 0.65 |
| -19.98 | 184.33 | 11 | 45 | 0.54 |
| -19.98 | 184.33 | 11 | 30 | 0.84 |
| -19.98 | 184.33 | 11 | 10 | 1.16 |
| -19.5 | 187.2 | 12 | 500 | 0.66 |
| -19.5 | 187.2 | 12 | 300 | 0.47 |
| -19.5 | 187.2 | 12 | 250 | 1.10 |
| -19.5 | 187.2 | 12 | 200 | 0.33 |
| -19.5 | 187.2 | 12 | 150 | 0.36 |
| -19.5 | 187.2 | 12 | 100 | 0.48 |
| -19.5 | 187.2 | 12 | 70 | 0.45 |
| -19.5 | 187.2 | 12 | 50 | 0.50 |
| -19.5 | 187.2 | 12 | 30 | 0.94 |
| -18.2 | 189.1 | LD_B | 500 | 0.34 |
| -18.2 | 189.1 | LD_B | 450 | 0.23 |
| -18.2 | 189.1 | LD_B | 400 | 0.41 |
| -18.2 | 189.1 | LD_B | 350 | 0.28 |
| -18.2 | 189.1 | LD_B | 300 | 0.43 |
| -18.2 | 189.1 | LD_B | 200 | 0.30 |
| -18.2 | 189.1 | LD_B | 150 | 0.38 |
| -18.2 | 189.1 | LD_B | 100 | 0.71 |
| -18.2 | 189.1 | LD_B | 80 | 0.39 |
| -18.2 | 189.1 | LD_B | 50 | 0.30 |
| -18.2 | 189.1 | LD_B | 30 | 0.52 |
| -18.2 | 189.1 | LD_B | 10 | 0.59 |
| -18.2 | 189.1 | LD_B | 5 | 0.71 |
| -18.4 | 194.1 | LD_C | 500 | 0.29 |
| -18.4 | 194.1 | LD_C | 450 | 0.29 |
| -18.4 | 194.1 | LD_C | 400 |  |
| -18.4 | 194.1 | LD_C | 350 | 0.25 |
| -18.4 | 194.1 | LD_C | 300 |  |
| -18.4 | 194.1 | LD_C | 250 | 0.16 |
| -18.4 | 194.1 | LD_C | 200 | 0.35 |
| -18.4 | 194.1 | LD_C | 150 | 0.24 |
| -18.4 | 194.1 | LD_C | 100 | 0.17 |
| -18.4 | 194.1 | LD_C | 50 | 0.16 |
| -18.4 | 194.1 | LD_C | 10 | 0.35 |
| -18.4 | 194.1 | LD_C | 5 | 0.39 |
| -18.42 | 197 | 14 | 500 | 0.59 |
| -18.42 | 197 | 14 | 450 | 0.28 |
| -18.42 | 197 | 14 | 400 | 0.34 |
| -18.42 | 197 | 14 | 350 | 0.29 |
| -18.42 | 197 | 14 | 300 | 0.27 |
| -18.42 | 197 | 14 | 250 | 0.49 |
| -18.42 | 197 | 14 | 200 | 0.27 |
| -18.42 | 197 | 14 | 150 | 0.31 |
| -18.42 | 197 | 14 | 100 | 0.32 |
| -18.42 | 197 | 14 | 80 | 0.31 |
| -18.42 | 197 | 14 | 30 | 0.21 |
| -18.42 | 197 | 14 | 5 | 0.46 |
| -18.27 | 200 | 15 | 500 | 0.48 |
| -18.27 | 200 | 15 | 450 | 0.40 |
| -18.27 | 200 | 15 | 400 | 0.26 |
| -18.27 | 200 | 15 | 250 | 0.25 |
| -18.27 | 200 | 15 | 200 | 0.40 |
| -18.27 | 200 | 15 | 150 | 0.47 |
| -18.27 | 200 | 15 | 100 | 0.28 |
| -18.27 | 200 | 15 | 80 | 0.37 |
| -18.27 | 200 | 15 | 50 | 0.34 |
| -18.27 | 200 | 15 | 30 | 0.20 |
| -18.27 | 200 | 15 | 10 | 0.31 |
| -18.27 | 200 | 15 | 5 | 0.31 |
